# Supplementary material for: Profile of MicroRNAs Associated with Death Due to Disease Progression in Metastatic Papillary Thyroid Carcinoma Patients
Source: Cancers (Basel). 2023 Jan 31;15(3):869. doi: 10.3390/cancers15030869 (PMC9913691; doi:10.3390/cancers15030869)
Supplement: Supplementary file 1 [file cancers-15-00869-s001.zip › cancers-2159251-supplementary.pdf]

Supplemenary Materials: Profile of MicroRNAs Associated with Death Due to Disease Progression in Metastatic Papillary Thyroid Carcinoma Patients

Ana Kober Leite , Kelly Cristina Saito, Thérèse Rachell Theodoro, Fátima Solange Pasini, Luana Perrone Camilo, Carlos Augusto Rossetti, Beatriz Godoi Cavalheiro, Venâncio Avancini Ferreira Alves, Luiz Paulo Kowalski, Maria Aparecida Silva Pinhal, Edna Teruko Kimura [3] and Leandro Luongo Matos

Table S1. Specific primer pairs for each sequence used in *TERT* promoter and *BRAF* mutation detection.

Mutation	Sequence of the primers	Annealing	Product length
TERT promoter (C228T and C250T)	PCR I<br>F: 5`-ACGAACGTGGCAGCGGCAG-3`<br>R: 5`-CTGGCGTCCCTGCACCCTGG-3`	60°C	474pb
	PCR II<br>F: 5`-CAGCGCTGCCTGAAACTC-3`<br>R: 5`-GTCCTGCCCCTTCACCTT-3`	60°C	163pb
BRAF^{T1799A}	PCR I<br>F: 5`-GACTCTAAGAGGAAAGATGAAGTA-3`<br>R: 5`-GATTTTTGTGAATACTGGGAACTATGA-3`	58°C	390bp
	PCR II<br>F: 5`-AAACTCTTCATAATGCTTGCTTGCCTG-3`<br>R: 5`-GGCCAAAAATTTAATCAGTGGA-3`	60°C	231bp

Table S2. Primers sequences for the studied microRNAs.

microRNA	Sequence
let-7b-5p	UGAGGUAGUAGGUUGUGGUU
let-7c-5p	UGAGGUAGUAGGUUGUAUGGUU
let-7d-5p	AGAGGUAGUAGGUUGCAUAGUU
let-7e-5p	UGAGGUAGGAGGUUGUAUAGUU
let-7f-5p	UGAGGUAGUAGAUUGUAUAGUU
let-7g-5p	UGAGGUAGUAGUUUGUACAGUU
let-7i-5p	UGAGGUAGUAGUUUGUGCUGUU
miR-1-3p	UGGAAUGUAAAGAAGUAUGUAU
miR-101-3p	UACAGUACUGUGAUAACUGAA
miR-10b-5p	UACCCUGUAGAACCGAAUUUGUG
miR-125a-5p	UCCUGAGACCCUUUAACCUGUGA
miR-129-5p	CUUUUUGCGGUCUGGGGCUUGC
miR-130b-3p	CAGUGCAAUGAUGAAAGGGCAU
miR-137-3p	UUAUUGCUUAAGAAUACGCGUAG
miR-138-2-3p	GCUAUUUCACGACACCAGGGUU
miR-138-5p	AGCUGGUGUUGUGAAUCAGGCCG
miR-141-3p	UAACACUGUCUGGUAAAGAUGG
miR-146b-3p	UGCCCUGUGGACUCAGUUCUGG
miR-146b-5p	UGAGAACUGAAUUCCAUAGGCU
miR-155-3p	CUCCUACAUAUUAGCAUUAACA
miR-16-5p	UAGCAGCACGUAAAUAUUGGCG
miR-17-3p	ACUGCAGUGAAGCACUUGUAG
miR-17-5p	CAAAGUGCUUACAGUGCAGGUAG
miR-181a-5p	AACAUUCAACGCUGUCGGUGAGU
miR-181b-5p	AACAUUCAUUGCUGUCGGUGGGU
miR-187-3p	UCGUGUCUUGUGUGCAGCCGG
miR-18a-5p	UAAGGUGCAUCUAGUGCAGAUAG
miR-191-5p	CAACGGAAAUCCCAAAAGCAGCUG

miR-199a-3p	ACAGUAGUCUGCACAUUGGUUA
miR-19a-3p	UGUGCAAAUCUAUGCAAAACUGA
miR-19b-3p	UGUGCAAAUCCAUGCAAAACUGA
miR-200a-3p	UAACACUGUCUGGGUAACGAUGU
miR-200b-3p	UAAUACUGCCUGGUAAUGAUGA
miR-200c-3p	UAAUACUGCCGGGUAAUGAUGGA
miR-203a-3p	GUGAAAUGUUUAGGACCACUAG
miR-205-5p	UCCUUCAUUCCACCGGAGUCUG
miR-20a-5p	UAAAGUGCUUAUAAGUGCAGGUAG
miR-21-5p	UAGCUUAUCAGACUGAUGUUGA
miR-214-3p	ACAGCAGGCACAGACAGGCAGU
miR-221-3p	AGCUACAUUGUCUGCUGGGUUUC
miR-222-3p	AGCUACAUCUGGCUACUGGGU
miR-29a-3p	UAGCACCAUCUGAAAUCGGUUA
miR-302c-3p	UAAGUGCUUCCAUGUUUCAGUGG
miR-30a-5p	UGUAAACAUCCUCGACUGGAAG
miR-30b-5p	UGUAAACAUCCUACACUCAGCU
miR-30c-5p	UGUAAACAUCCUACACUCUCAGC
miR-30d-5p	UGUAAACAUCCCCGACUGGAAG
miR-30e-3p	CUUUCAGUCGGAUGUUUACAGC
miR-30e-5p	UGUAAACAUCCUUGACUGGAAG
miR-31-5p	AGGCAAGAUGCUGGCAUAGCU
miR-34a-5p	UGGCAGUGUCUUAGCUGGUUGU
miR-34b-3p	CAAUCACUAACUCCACUGCCAU
miR-34c-5p	AGGCAGUGUAGUAGCUGAUUGC
miR-423-5p	UGAGGGGCAGAGAGCGAGACUUU
miR-429	UAAUACUGUCUGGUAAAACCGU
miR-455-3p	GCAGUCCAUGGGCAUAUACAC
miR-4788	UUACGGACCAGCUAAGGGAGGC
miR-483-3p	UCACUCCUCUCCUCCCGUCUU
miR-506-3p	UAAGGCACCCUUCUGAGUAGA
miR-654-3p	UAUGUCUGCUGACCAUCACCUU
miR-9-5p	UCUUUGGUUAUCUAGCUGUAUGA
miR-92a-3p	UAUUGCACUUGUCCCGGCCUGU
miR-98-5p	UGAGGUAGUAAGUUGUAUUGUU

Table S3. Variables not significant in the multivariate linear regression analyses.

Model		Beta In	t	Sig.	Partial Correlation	Collinearity Statistics Tolerance
1	*let-7c-5p*	−0.325[b]	−1.848	0.138	−0.679	0.348
	let-7e-5p	−0.161[b]	−1.130	0.322	−0.492	0.743
	let-7i-5p	0.223[b]	2.555	0.063	0.787	0.997
	miR-101-3p	0.294[b]	2.905	0.044	0.824	0.627
	miR-138-5p	−0.153[b]	−0.952	0.395	−0.430	0.635
	miR-16-5p	0.277[b]	2.230	0.090	0.744	0.580
	miR-181b-5p	−0.241[b]	−1.756	0.154	−0.660	0.602
	miR-191-5p	0.182[b]	0.923	0.408	0.419	0.425
	miR-19a-3p	0.125[b]	0.802	0.468	0.372	0.708
	miR-19b-3p	0.071[b]	0.381	0.723	0.187	0.560
	miR-200b-3p	−0.093[b]	−0.690	0.528	−0.326	0.996
	miR-200c-3p	0.039[b]	0.098	0.927	0.049	0.125
	miR-20a-5p	0.008[b]	0.027	0.980	0.013	0.220
	miR-29a-3p	0.140[b]	0.650	0.551	0.309	0.391
	miR-30a-5p	−0.041[b]	−0.237	0.824	−0.118	0.663
	miR-30b-5p	−0.184[b]	−1.000	0.374	−0.447	0.475
	miR-30c-5p	−0.235[b]	−1.402	0.234	−0.574	0.476
	miR-30d-5p	0.197[b]	1.927	0.126	0.694	0.996

	miR-31-5p	0.026[b]	0.180	0.866	0.089	0.976
	miR-34a-5p	0.217[b]	0.944	0.398	0.427	0.310
	miR-423-5p	−0.214[b]	−1.537	0.199	−0.609	0.650
2	*let-7c-5p*	−0.175[c]	−1.175	0.325	−0.561	0.266
	let-7e-5p	0.006[c]	0.044	0.968	0.025	0.466
	let-7i-5p	0.077[c]	0.398	0.717	0.224	0.218
	miR-138-5p	0.058[c]	0.397	0.718	0.223	0.375
	miR-16-5p	0.192[c]	2.784	0.069	0.849	0.505
	miR-181b-5p	−0.108[c]	−0.834	0.466	−0.434	0.418
	miR-191-5p	0.231[c]	4.442	0.021	0.932	0.418
	miR-19a-3p	−0.183[c]	−1.443	0.245	−0.640	0.315
	miR-19b-3p	−0.207[c]	−1.963	0.144	−0.750	0.339
	miR-200b-3p	−0.096[c]	−1.280	0.291	−0.594	0.996
	miR-200c-3p	−0.079[c]	−0.301	0.783	−0.171	0.121
	miR-20a-5p	−0.180[c]	−0.988	0.396	−0.495	0.195
	miR-29a-3p	0.036[c]	0.233	0.831	0.133	0.359
	miR-30a-5p	0.066[c]	0.569	0.609	0.312	0.584
	miR-30b-5p	−0.033[c]	−0.220	0.840	−0.126	0.372
	miR-30c-5p	−0.137[c]	−1.150	0.334	−0.553	0.421
	miR-30d-5p	0.006[c]	0.038	0.972	0.022	0.303
	miR-31-5p	0.022[c]	0.236	0.829	0.135	0.975
	miR-34a-5p	−0.101[c]	−0.476	0.667	−0.265	0.176
	miR-423-5p	−0.126[c]	−1.273	0.293	−0.592	0.566
3	*let-7c-5p*	0.003[d]	0.025	0.982	0.018	0.168
	let-7e-5p	−0.034[d]	−0.604	0.607	−0.393	0.451
	let-7i-5p	−0.057[d]	−0.661	0.576	−0.424	0.184
	miR-138-5p	0.030[d]	0.462	0.689	0.311	0.370
	miR-16-5p	−0.016[d]	−0.107	0.924	−0.076	0.075
	miR-181b-5p	−0.039[d]	−0.631	0.593	−0.407	0.377
	miR-19a-3p	−0.039[d]	−0.456	0.693	−0.307	0.205
	miR-19b-3p	−0.053[d]	−0.576	0.623	−0.377	0.173
	miR-200b-3p	−0.042[d]	−1.222	0.346	−0.654	0.832
	miR-200c-3p	0.134[d]	1.470	0.279	0.721	0.098
	miR-20a-5p	0.045[d]	0.396	0.730	0.270	0.122
	miR-29a-3p	0.068[d]	1.351	0.309	0.691	0.353
	miR-30a-5p	−0.036[d]	−0.655	0.580	−0.420	0.450
	miR-30b-5p	0.006[d]	0.090	0.936	0.064	0.363
	miR-30c-5p	0.001[d]	0.017	0.988	0.012	0.271
	miR-30d-5p	−0.082[d]	−1.566	0.258	−0.742	0.276
	miR-31-5p	0.009[d]	0.225	0.843	0.157	0.969
	miR-34a-5p	−0.120[d]	−2.440	0.135	−0.865	0.176
	miR-423-5p	0.112[d]	2.088	0.172	0.828	0.185

b. Predictors in the Model: (Constant), miR-17-5p; c. Predictors in the Model: (Constant), miR-17-5p, miR-101-3p; d. Predictors in the Model: (Constant), miR-17-5p, miR-101-3p, miR-191-5p.
